# Efficacy of a Single-Task ERP Measure to Evaluate Cognitive Workload During a Novel Exergame

**DOI:** 10.3389/fnhum.2021.742384

**Published:** 2021-09-08

**Authors:** Usman Ghani, Nada Signal, Imran Khan Niazi, Denise Taylor

**Affiliations:** ^1^Rehabilitation Innovation Centre, Auckland University of Technology, Auckland, New Zealand; ^2^Department of Health Science and Technology, Aalborg University, Aalborg, Denmark; ^3^Centre for Chiropractic Research, New Zealand College of Chiropractic, Auckland, New Zealand

**Keywords:** cognitive workload, exergame, electroencephalogram, event-related potentials, rehabilitation

## Abstract

This study aimed to validate the efficacy of single-task event-related potential (ERP) measures of cognitive workload to be implemented in exergame-based rehabilitation. Twenty-four healthy participants took part in a novel gamified balance task where task-irrelevant auditory tones were presented in the background to generate ERPs in the participants’ electroencephalogram (EEG) as a measure of cognitive workload. For the balance task, a computer-based tilt-ball game was combined with a balance board. Participants played the game by shifting their weight to tilt the balance board, which moved a virtual ball to score goals. The game was manipulated by adjusting the size of the goalposts to set three predefined levels of game difficulty (easy, medium, and hard). The participant’s experience of game difficulty was evaluated based on the number of goals scored and their subjective reporting of perceived difficulty. Participants experienced a significant difference in the three levels of task difficulty based on the number of goals scored and perceived difficulty (*p* < 0.001). Post hoc analysis revealed the lowest performance for the hardest level. The mean amplitude of the N1 ERP component was used to measure the cognitive workload associated with the three difficulty levels. The N1 component’s amplitude decreased significantly (*p* < 0.001), with an increase in the task difficulty. Moreover, the amplitude of the N1 component for the hard level was significantly smaller compared to medium (*p* = 0.0003) and easy (*p* < 0.001) levels. These results support the efficacy of the N1 ERP component to measure cognitive workload in dynamic and real-life scenarios such as exergames and other rehabilitation exercises.

## Introduction

In rehabilitation, the level of cognitive workload for an individual patient is, in part, dependent on the task difficulty. Task difficulty is related to variables such as the number of repetitions of the task and the intensity of the task or how *hard* the person is working (Brody, 2012). These variables are important for clinicians to consider when setting rehabilitation programs and lead the clinician to determine how challenging each rehabilitation task is for the individual and the optimal number of repetitions and intensity required to achieve good rehabilitation outcomes for each patient. In other fields a number of subjective procedures have been developed for measuring cognitive workload. In particular, the Cooper–Harper Scale (Cooper and Harper, 1969), the Subjective Workload Assessment Technique (Reid and Nygren, 1988), and the NASA-TLX are widely used (Hill et al., 1992; Rubio et al., 2004; Hart, 2006). However, these subjective measures are insensitive to cognitive workload changes that occur *during* the task or rehabilitation session (Eggemeier, 1988; Deeny et al., 2014). Currently, there is no objective measure sensitive enough to evaluate cognitive workload during the performance of a rehabilitation task. Therefore, this study proposed an electroencephalogram (EEG) based paradigm to measure cognitive workload during rehabilitation.

EEG has the potential to measure cognitive workload with a high temporal resolution while allowing freedom of movement during data collection, thus facilitating adaptability to clinical, operational, or real-world settings (Kruse, 2007; Lan et al., 2007; Casson et al., 2008; Seneviratne et al., 2013). Remarkably, although efforts to use measures of cognitive workload such as event-related potentials (ERPs) in EEG are increasingly abundant in the literature for several real-life tasks (Kramer et al., 1995; Suzuki et al., 2005; Allison and Polich, 2008; Miller et al., 2011; Causse et al., 2015; Takeda et al., 2016), ERP measures of cognitive workload have not been adapted and applied to the field of rehabilitation. Our previous study evaluated the cognitive workload in three predefined difficulty levels (easy, medium, and hard) during a custom-made visuomotor task (Ghani et al., 2020a). The task used was a tilt-ball game (played on an iPad with participants sitting on a chair). The study involved 25 healthy young adults (age range 20–30 years). There were three predefined difficulty levels, and the target was to move the ball (by tilting an iPad) into highlighted goals while avoiding the obstacles. Goals scored, collisions with moving obstacles, and subjective ratings were used as performance measures. The results showed a significant decrease in the N1 ERP component with increased task difficulty. Similarly, both behavioral measures showed significant effects of task difficulty. For example, goals scored were significantly decreased, and subjective ratings were significantly increased when the task difficulty was increased from easy to medium to hard.

The current study aimed to validate the same approach to evaluating cognitive workload during rehabilitation settings. We developed a custom-made exergame with three predefined difficulty levels. Exergames incorporate exercises into on-screen computer games or use in clinical rehabilitation settings (Fitzgerald et al., 2010; Gil-Gómez et al., 2011; Harvey and Ada, 2012; van den Berg et al., 2016). The main idea behind introducing exergames into rehabilitation is to motivate and enhance engagement in rehabilitation (van den Berg et al., 2016). The exergame used in this study had two parts (1) the cognitive (tilt-ball game) and (2) the physical (balance board) components. We kept the challenge in the balance component of the task constant and to a minimum to ensure that the participants were preferentially focused on the cognitive component (tilt-ball game). Similar to our previous study, the current study utilized task-irrelevant auditory stimuli to generate ERP components, and no instructions for these stimuli were given to the participants. Hence, these stimuli were expected to consume involuntary attention orienting response highlighted by the early ERP components (N1, P1, P2) (Näätänen and Michie, 1979; Ghani et al., 2020a, b).

Out of these early ERP components, the N1 ERP component is strongly associated with stimulus filtering and involuntary attention orienting (Näätänen and Michie, 1979; Takeda et al., 2016; Ghani et al., 2020a). The N1 ERP component is also considered to mark stimulus detection and perhaps later stages of sensory processing in conjunction with later ERP components (Fogarty et al., 2020). These properties make the N1 ERP component the most suitable to look at during a task-irrelevant auditory ERP paradigm. Therefore, we selected the N1 ERP component’s amplitude concerning cognitive workload and hypothesized that the N1 ERP component’s amplitude would decrease with the increased cognitive workload.

## Materials and Methods

### Participants

An a priori power analysis was conducted using G^∗^Power3 (Faul et al., 2009) with previously reported effect size ($\eta_{\rho }^{2}$ = 0.264) (Ghani et al., 2020a), power (β = 0.8), and significance level (α = 0.05). A total of twenty-four healthy young adults (11 females, age range: 20–30, mean age: 25 ± 3.4) were recruited via advertisements through university networks and word of mouth. People with a neurological disorder, hearing loss, recent head injury, or metal implants were excluded from the study. Participants were advised to avoid caffeine before the experiment and asked about their caffeine intake for the day on arrival. All the participants signed a written informed consent before the experiment and received a $20 gift voucher.

### Task

**FIGURE 1:**
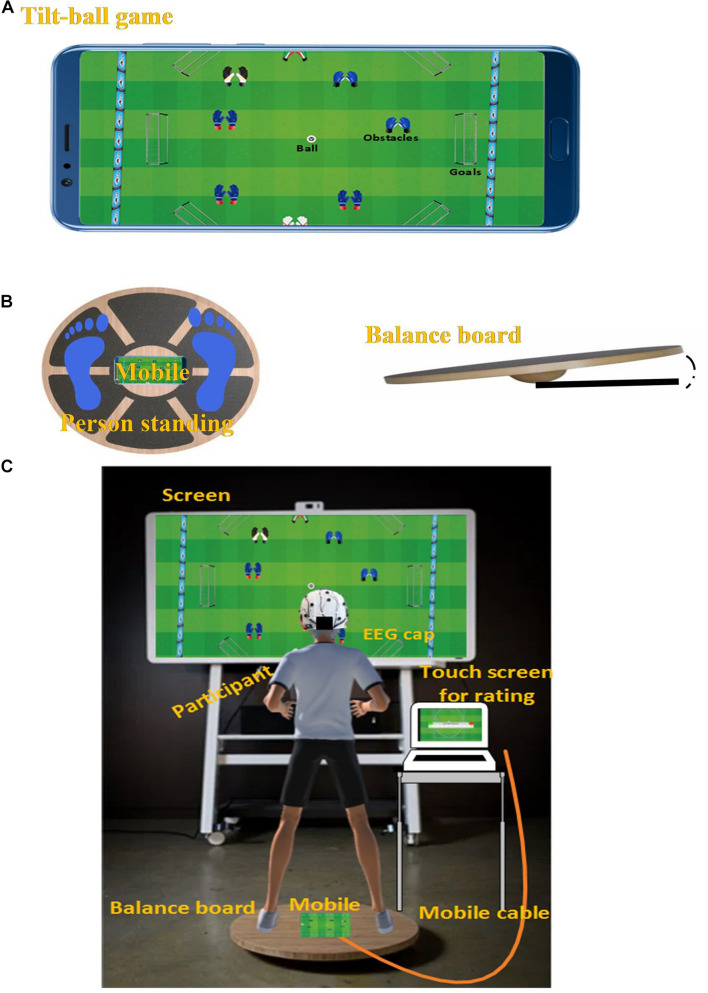
Panel **(A)** shows a tilt-ball game on an android device. Panel **(B)** shows a balance board with a tilt angle and top view of a person standing on the balance board. Panel **(C)** shows the complete study setup.

The exergame rehabilitation task involved playing a tilt-ball game via a balance board. Participants stood on a balance board which could tilt in multiple directions up to an angle of ten degrees. While standing on the board, the participant could control the tilt direction and angle by moving their center of mass. The custom designed tilt-ball game (see Figure 1A) was installed on an android phone embedded in the center of a balance board, as shown in Figure 1B. The participant tilted the balance board, and consequently the phone, to control the ball within the tilt ball game. The tilt-ball game was projected from the phone to a screen in front of the participants. This complete setup is shown in Figure 1C.

The tilt-ball game had eight different goalposts, a soccer ball, and a moving obstacle. One of the eight goalposts was highlighted randomly, and the task was to move the soccer ball into the highlighted goalpost by tilting the balance board. The participant scored one point for each goal. The absolute difficulty of the task was manipulated by adjusting the size of the goalposts. Three absolute difficulty levels (easy, medium, and hard) were predefined. The easy level had a large goalpost (1 unit long) as compared to medium (0.8 unit long) and hard (0.6 unit long) levels.

### Procedure

**FIGURE 2:**
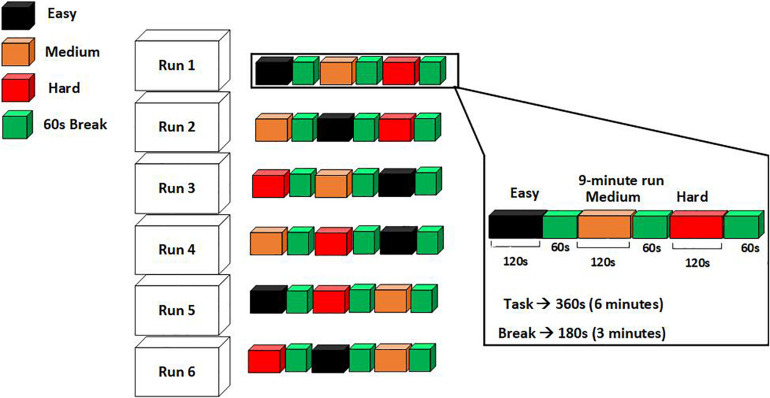
Figure shows the procedure of presenting three difficulty levels in random order during six separate runs.

After participants had provided written informed consent, they undertook six minutes of practice to familiarize themselves with the exergame. They were then prepared for EEG recording (section “EEG Data Collection and Processing”). Data collection was undertaken in six separate runs of nine minutes each. In each run, three predefined difficulty levels (easy, medium, and hard) were presented in a random order (randomization was done using a MATLAB code), where each level lasted for two minutes. After each two-minute block, a one-minute break was given. In this break time, the participants were instructed to sit on a chair and asked to subjectively rate the task difficulty of the block on a numeric scale (1 = “Very easy” to 10 = “very hard”). This presentation is shown in Figure 2, with three difficulty levels highlighted in different colors. The participants experience of task difficulty was evaluated in two ways; (1) the number of goals scored during each level and (2) the subjective rating of perceived task difficulty.

During the task, 1,000 Hz tones (100 ms duration, 10 ms rise/fall time, 95 dB SPL) were presented over a pair of speakers placed about 50 cm behind the participant. These tones were presented in the background, and no instruction was given to the participant about the auditory stimuli. According to the literature, the interstimulus interval can affect the amplitude of ERP components (Gonsalvez et al., 2007). Therefore, based on the study of Allison and Polich (2008), the auditory tone interstimulus interval was varied randomly between 6 to 10 s. There were 45 tones presented during each run, with 270 tones presented to a single participant while performing the task.

### EEG Data Collection and Processing

The EEG data was recorded using a 64 channel Brainwave EEG cap with a REFA amplifier (TMSi, Twente, Netherlands) at a sampling rate of 2,048 Hz. EEG data was recorded from all 64 scalp sites according to a 10–20 electrode system (Homan, 1988). The ground electrode was placed at AFz, and both mastoids (M1 and M2) were used as a reference for the recording. The impedance of all the electrodes was kept below 10 kΩ. The online filter settings were DC −100 Hz, where a 50 Hz notch filter was also used during the recording of raw data. The raw EEG data were preprocessed offline using EEGLAB (version 14.1.1) (Delorme and Makeig, 2004) and ERPLAB (version 6.1.4) (Lopez-Calderon and Luck, 2014) running on MATLAB (2015b) (The MathWorks, Inc, Natick, MA, United States).

The PREP pipeline (version 0.55.1) (Bigdely-Shamlo et al., 2015) was used to remove and interpolate bad channels, remove line noise, and find the average reference. Then the data was high pass filtered at 1 Hz before independent component analysis (ICA). ICA and IClabel (Pion-Tonachini et al., 2019) were used to visually remove noisy components such as eyeblinks or other muscle artifacts. The data was then bandpass filtered at (0.05–30 Hz). Following preprocessing, epochs were extracted from −200 to 1,000 ms to the stimulus and were baseline corrected using the pre-stimulus period. The epochs obtained were then subjected to the ERPLAB artifact detection algorithm of moving window threshold (Lopez-Calderon and Luck, 2014). A 200 ms window width and a 100 ms step were defined with a threshold of ±100 μV. The epochs in which the signal exceeded ±100 μV on any channel were rejected.

The grand-average ERP waveform for each predefined difficulty level (collapsed across all runs) was calculated. The latency window of the N1 ERP component for all three predefined difficulty levels (easy, medium, and hard) was defined as previously reported (Ghani et al., 2020a). The reported method suggests placing a narrow time window around the peaks in the grand average ERP waveform of the Cz electrode. The grand averaged ERP waveform was obtained by averaging the waveform of three levels (easy, medium, and hard). This ERP waveform was then used to mark narrow time windows across three prominent peaks. The latency window for the N1 component obtained from this method was 150–230 ms for three midline electrodes (Fz, Cz, and Pz). This latency information was provided to ERP measurement tool (Lopez-Calderon and Luck, 2014) to extract amplitude of the N1 component. After all the pre-processing steps on average 10 ± 5 epochs were rejected per level for each participant. However, the number of epochs across each level (easy, medium, and hard) were kept constant.

### Statistical Analysis

The statistical analysis was divided into two phases (1) analysis of performance data and (2) analysis of physiological data. Two separate repeated measures analysis of variance (ANOVA) tests with main terms of predefined difficulty level (easy, medium, and hard) and the measures of experienced difficulty (goals scored and subjective rating of difficulty) were used for the analysis of performance data. The goals scored and ratings of difficulty for each level were averaged across six runs for each participant. For the physiological data, a 3 × 3 (level × channels) repeated measures ANOVA with main terms of predefined difficulty level (easy, medium, and hard) and the measure of cognitive workload (mean amplitude of N1) was used. The data was then rearranged by averaging across three electrodes for each level. Finally, the data was subjected to a *post-hoc* pairwise comparison of each level (easy, medium, and hard). The Bonferroni adjusted values are reported for all *post-hoc* comparisons. Conventional degrees of freedom are reported throughout the results. Additionally, effect sizes were reported when required. For *post-hoc* correlation analysis, we looked at the correlations between the change in outcome measures (Easy–Hard) using Pearson’s correlation. The outcome measures used in this analysis were behavioral measures (goals scored, subjective ratings) and physiological measures (the N1 ERP component). The amplitude of the N1 ERP component was the average taken from three midline electrodes (Fz, Cz, and Pz). The correlation between the change in the amplitude of the N1 ERP component and change in goals scored, the change in the amplitude of the N1 ERP component and change in subjective ratings, and the change in subjective ratings and change in goals scored, was examined separately.

## Results

Task performance parameters (goals scored and difficulty ratings) were used to measure perceived difficulty to ensure that the participants had experienced three predefined levels of task difficulty (easy, medium, and hard). The N1 ERP component was then used to measure cognitive workload associated with the three predefined levels of task difficulty. Finally, to look at the effect of increasing task difficulty on attentional demands a correlation analysis between behavioral and physiological measures was conducted.

### Behavioral Results

**FIGURE 3:**
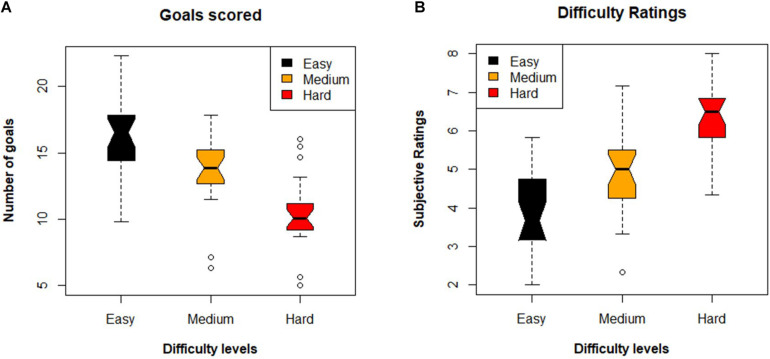
Panel **(A)** shows the boxplots of the mean goal scored during each difficulty level. Panel **(B)** shows the boxplots of the subjective difficulty ratings. The has outliers so whiskers represent one and a half times the interquartile range (1.5 × IQR).

Both measures of perceived difficulty goals scored *F*(2,46) = 26.9, *p* < 0.001, $\eta_{\rho }^{2}$ = 0.438 and difficulty ratings *F*(2,46) = 32.2, *p* < 0.001, $\eta_{\rho }^{2}$ = 0.483 showed that the participants experienced significant differences in the three levels of task difficulty. *Post-hoc* analysis revealed that the number of goals scored during the easy level was significantly greater than goals scored during the medium [*t*(69) = −3.29, *p* < 0.005] and hard [*t*(69) = 7.32, *p* < 0.001] levels, respectively. Similarly, the goals scored during medium level were significantly greater than goals scored during hard level [*t*(69) = 4.03, *p* < 0.001]. For the second measure of perceived difficulty, the subjective ratings given by the participants to the easy level were significantly lower than medium [*t*(69) = −3.64, *p* = 0.001] and hard [*t*(69) = −8.01, *p* < 0.001] levels. Similarly, the medium level received a lower rating than the hard level [*t*(69) = −4.37, *p* < 0.001]. These results are shown in Figure 3.

### Electrophysiological Measures

**FIGURE 4:**
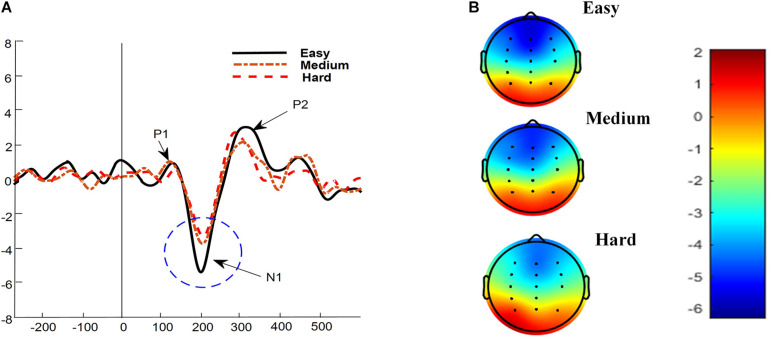
Panel **(A)** shows a grand average ERP waveform of 24 participants: P1, P2, and N1 are evident. Panel **(B)** shows scalp maps of the N1 component for three levels of cognitive workload.

Figure 4A illustrates the grand average ERPs for each predefined difficulty level (easy, medium, and hard). The P1, P2, and N1 components are evident, and the N1 ERP component is highlighted using a dotted circle. In the previous study, the N1 component’s amplitude showed a significant cognitive workload change (Ghani et al., 2020a). Therefore, in this study, the N1 ERP component’s amplitude from three midline channels (Fz, Cz, and Pz) was evaluated as a measure of cognitive workload. There was no level channel interaction *F*(4,92) = 0.209, *p* = 0.933, $\eta_{\rho }^{2}$ = 0.005, and the statistical analysis revealed a main effect for predefined difficulty levels (easy, medium, and hard) for the mean amplitude of the N1 component *F*(2,46) = 94.6, *p* < 0.001, $\eta_{\rho }^{2}$ = 0.471. The effect of channel was also not significant *F*(2,46) = 1.026, *p* = 0.280, $\eta_{\rho }^{2}$ = 0.012. The N1 ERP component exhibits a frontocentral scalp distribution (Parasuraman and Beatty, 1980), shown in Figure 4B for all three levels of predefined difficulty (easy, medium, and hard).

Post hoc analysis with respective means, confidence intervals, and Cohen’s d effect size is shown in Table 1. Post hoc analysis revealed that for the N1 component, the mean amplitude during the hard level was significantly lower than during the easy [Hard < Easy, *t*(69) = −3.84, *p* < 0.001] and medium levels [Hard < medium, *t*(69) = −2.28, *p* = 0.001]. Similarly, the medium level’s mean amplitude was significantly lower than the easy level [Medium < Easy, *t*(69) = −6.12, *p* < 0.001].

**TABLE 1:** Table shows the mean values and effect sizes from the *post-hoc* comparison.

ERP component	Level	Mean	95% confidence interval		Pairwise comparison	*p*-value	Effect-size Cohen’s (*d*)
			Lower	Upper			
N1	Easy	−3.64	−3.88	−3.40	Easy–medium	<0.001	1.206
	Medium	−2.73	−2.97	−2.49	Easy–hard	<0.001	1.391
	Hard	−2.31	−2.55	−2.07	Medium–hard	0.0003	0.531

### Correlation Between Electrophysiological Measures and Performance Measures

The change in the physiological measure (the N1 ERP component) correlated significantly with the change in the number of goals scored [*r*(22) = 0.407, *p* = 0.049] as the difficulty increased from easy to hard. This suggests that as the performance difference increased, the difference between the amplitude of the N1 component between two difficulty levels also increased. On the other hand, the correlation between the change in the N1 ERP component and the change in subjective rating was not significant [*r*(22) = 0.224, *p* = 0.293]. Change in both behavioral measures such as goals scored and subjective ratings were correlated [*r*(22) = 0.642, *p* < 0.001], highlighting the consistency between the performance difference and subjective ratings difference.

## Discussion

The present study was designed to assess a single-task ERP method of evaluating cognitive workload to determine the possibility of using this method during rehabilitation. We intended to evaluate the cognitive workload associated with a novel exergame. Behavioral measures of task difficulty were recorded along with the EEG data. Behavioral results show that the performance of the participants decreased with an increase in task difficulty. On the physiological level, the amplitude of the N1 ERP component decreased significantly with an increase in task difficulty. These results were similar to those we obtained in our previous study (Ghani et al., 2020a) and were also in line with previous literature (Kramer et al., 1995; Suzuki et al., 2005; Combs and Polich, 2006; Muller-Gass and Schroger, 2007; Muller-Gass et al., 2007; Deeny et al., 2014). These findings validated our single task ERP paradigms’ efficacy to evaluate cognitive workload during rehabilitation settings.

According to the literature, the most basic tasks in psychological research are composed of different component operations (Posner and Raichle, 1994). Some of these component operations are more cognitive in nature, and others are more motoric (e.g., the tilt-ball game in this study compared to standing on a balance board). In the cognitive load theory, there are three types of cognitive workloads (1) intrinsic (task difficulty), (2) extraneous (depends on external parameters), and (3) germane (depends on working memory). Therefore, the task difficulty alone cannot define cognitive workload (Sweller, 1988). We have argued that an increase in the difficulty of the cognitive component of our task (requiring participants to score goals in smaller goalposts) imposed a combination of three cognitive workloads and induced participants to allocate more attention to the tilt-ball game. This shift of attention varied with the cognitive task difficulty; for example, more attention was given to the tilt-ball game as the cognitive component of the difficulty varied from easy to medium to hard. In this study, the proposed relationship of the change in cognitive task difficulty and attention was validated by the correlation between the change in the N1 ERP component and the change in number of goals scored as the task difficulty increased from easy to hard. For example, as the task difficulty increased, the difference in goals scored increased, more attention was likely given to the task in compensation, affecting the amplitude of the task-irrelevant auditory evoked N1 ERP component.

In this study, the N1 ERP component was selected as a measure of cognitive workload based on two possible reasons (1) the neural generators of the N1 ERP component and (2) properties of the N1 ERP component. The N1 generators are located mainly in the superior temporal plane, including the primary and secondary auditory cortices and auditory association areas (Näätänen and Picton, 1987; Lü et al., 1992; Pantev et al., 1995; Woods, 1995). The auditory association area is known to mediate auditory and visual workload [for review, see Calvert (2001)]. The finding that the auditory evoked N1 ERP component was significantly modulated by the cognitive workload imposed by the tilt-ball game suggests that the auditory association area is linked with a cross-modal capacity limit. Another supportive explanation is based on the generic properties of the N1 ERP component. As suggested by Dien et al. (1997); Picton et al. (1999); Grau et al. (2007), the N1 may also have sources in the frontal lobe, supporting links between the N1 and attention (Näätänen and Picton, 1987; Giard et al., 1994). Therefore, this association of the N1 ERP with both the cross-modal capacity limit and attention makes it a critical component in measuring cognitive workload using task-irrelevant auditory probes.

To date, there are no objective measures of the cognitive workload associated with any rehabilitation task, with health care practitioners relying on patient self-report. This study represents the first attempt to objectively quantify cognitive workload in rehabilitation settings, and the results are promising to investigate such methods. The N1 ERP component exhibited significant cognitive workload effects illustrating the inverse relationship between ERP (generated by task-irrelevant stimuli) amplitude and task difficulty. This paradigm is easily adaptable to research on various rehabilitation tasks where the cognitive workload is relevant. Wireless EEG caps used with this paradigm can enable real-time and offline EEG analysis for ecologically valid movements during various rehabilitation tasks. An additional advantage of the approach presented here is the sensitivity of the information acquired through a small number of electrodes. Although 64 channels of EEG data were obtained in this study, the results could have been obtained using only three midline electrodes (Fz, Cz, and Pz) with a ground and a reference (Ghani et al., 2020a).

The current study was limited to healthy participants and was conducted using an exergame. Future efforts will extend to patient populations and be adapted to other rehabilitation tasks. The use of a traditional averaged ERP paradigm limits the implementation of this research into rehabilitation settings, but it provides essential insights into how cognitive workload affects ERPs in rehabilitation-like settings. These insights can then be used with more advanced techniques such as single-trial detection of ERPs (Jung et al., 2001) to implement this research in actual rehabilitation settings. Another advantage of this research in its current form is that it can be used to validate the clinical efficacy of available rating scales used in rehabilitation. The use of attentional reserve-based paradigm (ERPs) of assessing cognitive workload also has broad adaptability for comparing different tasks and strategies in various rehabilitation settings. Furthermore, combining the current paradigm with more sophisticated approaches such as source localization (Jatoi et al., 2014) and obtaining data from more channels (Michel and Brunet, 2019) can simultaneously address task difficulty, regional activation, and functional communication between different cortical regions (Rietschel et al., 2012) to examine the sensory, motor, and cognitive demands.

## Conclusion

This study aimed to examine the efficacy of using ERPs as an outcome measure for cognitive workload in rehabilitation settings, specifically during exergames. The amplitude of the task-irrelevant stimuli generated N1 ERP component decreased significantly with an increase in task difficulty. This decrease in the amplitude of the N1 ERP component can be used to evaluate the cognitive workload of a rehabilitation task objectively. The current study examined only an exergame-based task in healthy participants, which requires replication in patients and adaptation to other rehabilitation settings. However, this single-task ERP approach with task-irrelevant stimuli is adaptable to various rehabilitation tasks as an objective outcome measure of cognitive workload.

## Data Availability Statement

The original contributions presented in the study are included in the article/supplementary material, further inquiries can be directed to the corresponding author/s.

## Ethics Statement

The studies involving human participants were reviewed and approved by Auckland University of Technology Ethics Committee (AUTEC). The patients/participants provided their written informed consent to participate in this study.

## Author Contributions

UG designed and performed the experiments with consultation from NS, DT, and IN. UG derived the models and analyzed the data. IN assisted with data preprocessing and cleaning. UG wrote the manuscript in consultation with NS and DT. All authors contributed to the article and approved the submitted version.

## Conflict of Interest

The authors declare that the research was conducted in the absence of any commercial or financial relationships that could be construed as a potential conflict of interest.

## Publisher’s Note

All claims expressed in this article are solely those of the authors and do not necessarily represent those of their affiliated organizations, or those of the publisher, the editors and the reviewers. Any product that may be evaluated in this article, or claim that may be made by its manufacturer, is not guaranteed or endorsed by the publisher.
